# Adherence, perceptions and knowledge of an HIV PMTCT programme: A mother-baby pair study

**DOI:** 10.4102/sajhivmed.v26i1.1648

**Published:** 2025-01-28

**Authors:** Sthembiso Mabuka, Mygirl P. Lowane, Tintswalo V. Nesengani, Thembi V. Simbeni

**Affiliations:** 1 Department of Public Health, School of Health Care Sciences, Sefako Makgatho Health Sciences University, Ga-Rankuwa, South Africa; 2 Department of Nursing Science, Faculty of Health Sciences, University of Pretoria, Pretoria, South Africa

**Keywords:** adherence, perceptions, HIV, HIV-positive women, knowledge, mother-baby pairs, PMTCT programme, seroconversion

## Abstract

**Background:**

Prevention of mother-to-child transmission (PMTCT) programmes are designed to prevent HIV transmission to infants and children. Despite efforts to achieve this goal, several factors continue to pose challenges.

**Objectives:**

To investigate the level of adherence, perceptions, knowledge, and factors associated with adherence to the PMTCT programme in primary healthcare facilities.

**Method:**

A descriptive cross-sectional study design and quantitative research approach was used, and clinical records were reviewed to determine the prevalence of seroconverted babies of mothers enrolled in a PMTCT programme for the past 2 years in the community healthcare centres. Bivariate and multivariate logistic regression analyses were performed.

**Results:**

A total of 341 mother-baby pairs were recruited and took part in the study. Most women (263; 77%), perceived that a pregnant woman living with HIV can transmit the virus to her unborn baby. The following factors were independently associated with non-adherence: being unmarried, the period of maternal HIV diagnosis and initiation on antiretroviral therapy, unsuppressed viral load results, missed clinic appointments, side effects, and getting tired of taking HIV medication.

**Conclusion:**

This study investigated adherence to and perceptions of all components of the PMTCT programme by pregnant and breastfeeding women in primary healthcare facilities. Despite the significant progress made, maternal and paediatric HIV pandemic pose a challenge to the PMTCT services. There is a need for follow-up research to monitor the ongoing adherence to the PMTCT programme and its long-term impact in reducing the rate of transmission of HIV in mothers.

**What this study adds:** We identified factors associated with non-adherence to the PMTCT programme by pregnant and breastfeeding women, which could be used to target interventions.

## Introduction

HIV infections and AIDS are devastating diseases.^1,2^ The global prevalence of HIV/AIDS has rapidly increased from 9 to 37 million people in three decades, with an estimated 1.5 million pregnant women.^2,3,4^ In South Africa, women over the age of 19 years represent 5 million inhabitants,^5^ and seven out of eight new infections go unreported, with 95% of pregnant women receiving maternal care and antiretroviral therapy (ART) in public health facilities.^2,3^ Eliminating the transmission of HIV, to eradicate maternal-to-child HIV transmission (MTCT), is an important health service recommended for mothers with HIV.^6^ Over the past two decades, various global health programmes and interventions that include the provision of ART have been implemented to prevent the transmission of HIV from mother to child;^7^ however, the utilisation of the services remains low.^8^ In the absence of preventive measures, 90% of all global HIV infections of children occur as a result of mother-to-child transmission (MTCT).^9^

Recently, the world community committed to eradicate MTCT by 2030 or, if not possible, to less than 5% in breastfeeding countries and less than 2% in non-breastfeeding countries.^10^ A comprehensive approach was recommended by the World Health Organization (WHO), following four key pillars to improve the results in prevention of mother-to-child transmission (PMTCT): primary prevention of HIV infection, prevention of unwanted pregnancies in HIV-infected women, prevention of HIV transmission from infected mothers to their children, and care of HIV-infected mothers and their children.^11^ Other services include antenatal care (ANC), provision of ART for women and children living with HIV, prophylaxis for unaffected exposed babies, and infant feeding counselling.^12,13^

The WHO established a global HIV prevention strategy, estimating that 15% – 45% of children with HIV may be infected vertically.^14^ The cumulative vertical transmission rate by 18 months of age increases to 4.3%, and the largest proportion can reach 80% globally.^15^ This transmission occurred during the first 6 months of breastfeeding.^15^ Despite progress in eliminating MTCT, women are facing challenges in participating in the programme for various reasons, including delays in presentations in ANC health facilities, and poor health literacy.^16^ Lack of knowledge is one of the obstacles to the successful implementation of PMTCT programmes.^17^ Good adherence to the programme has remarkably reduced HIV-associated child mortality, and significantly reduced MTCT in all settings.^18^ Failure to adhere to the PMTCT programme has been a challenge in decreasing vertical transmission of HIV and associated mortality and morbidity in sub-Saharan African (SSA) countries.^11^

Despite the many successes of the PMTCT programme, transmission rates remain high in many parts of the world.^19^ In South Africa, efforts to achieve eradication of MTCT are challenged by several factors, including an extremely high maternal HIV seroprevalence of 30%.^20^ As a result, elimination of vertical transmission remains difficult.^14,20^ This observation is supported by the studies conducted in South Africa, which found that there are still babies seroconverting to HIV-positive during pregnancy or breastfeeding from mothers enrolled on the PMTCT programme.^12,21^ Assessing adherence to the PMTCT programme among mothers is essential. Therefore, the aim of the study is to investigate the levels of compliance, perceptions, knowledge, and factors related to compliance to the PMTCT programme among pregnant and breastfeeding women living with HIV.

## Research methods and design

### Study design

The study was carried out in pairs of mothers and babies. A descriptive cross-sectional study design and quantitative research approach was used, and clinical records were reviewed to evaluate the prevalence of seroconverted babies of mothers enrolled in PMTCT programmes during pregnancy and breastfeeding. A quantitative descriptive approach was used to determine the level of commitment, and factors affecting commitment to the PMTCT programme during pregnancy and breastfeeding among women.

### Setting

The study was conducted in Soweto, Johannesburg. Soweto is a semi-urban area surrounded by informal settlements in the southwest of Johannesburg in Gauteng province. Soweto covers Region D, with an estimated population of 1.9 million residents. Twenty-eight healthcare facilities within the region deliver services such as PMTCT services, HIV/AIDS counselling, testing, and ANC. The study was conducted in four community healthcare centres with a high volume of HIV-positive women on the PMTCT programme. The four selected facilities have a higher influx of women registered on the PMTCT programme than others.

### Study population and sampling strategy

The target population for this study consisted of HIV-positive postnatal and breastfeeding mother-baby pairs in the PMTCT programme at the selected public health facilities. The researcher purposely selected four healthcare facilities by considering the high number of HIV-positive postnatal and breastfeeding women enrolled on the PMTCT programme.

The desired sample size was estimated using a Raosoft sample size calculator, considering a 95% confidence interval (CI), 5% margin of error, and 87.1% level of ART adherence. A 10% buffer of respondents was included in the sample size to safeguard the possibility of a low or non-response rate. From the estimated population of 3500, the estimated sample size was 382. A non-probability convenient sampling technique was used to select the study respondents and the clinical records of their babies from the population attending the ANC in the selected facilities, until the required sample size was reached. Convenient sampling includes individuals who are most accessible and readily available to the researcher.^22^ However, due to field-related unforeseen challenges, only 341 (89%) respondents from the estimated sample size agreed to participate in this study.

Mothers living with HIV who had been enrolled in a PMTCT programme for the past 2 years, together with the road to health booklets (medical record) of their babies to determine the prevalence of children infected with HIV while on the PMTCT programme, were included in this study. Postnatal mothers who had lost their baby at the time of data collection and those who did not give consent to take part were excluded from this study.

### Recruitment and data collection

To ensure anonymity of recruitment, the researcher requested the supervisor of the healthcare facility or healthcare provider to inform eligible respondents of the study and to contact the researcher if they were interested in receiving more information in a private consultation room. The researchers worked closely with the interested respondent, after which they completed their consultations. The respondents were issued with a brochure containing information about the purpose of the study. When mothers agreed to participate, a researcher electronic questionnaire was used to collect data on the level of adherence to the PMTCT programme and its associated factors during pregnancy and breastfeeding. Data collection tools for mothers were divided into three sections, namely: section A, which covered the mother’s socio-demographic data; section B, which addressed the level of women’s adhesion to the PMTCT programme during pregnancy and breastfeeding; and section C, which determined the factors (knowledge and perceptions) that affect the non-compliance to the PMTCT programme during pregnancy and breastfeeding. The questionnaire was designed and modified variables guided by various studies relating to the compliance to the PMTCT programme and its associated factors,^10,22^ and it was also administered in English, Sesotho, and IsiZulu, depending on the language used or understood by the respondents. Each interview took approximately 15–25 minutes. Moreover, data were collected from baby clinic cards after the main interview, and the researchers used checklists to collect information on the prevalence of seroconverted babies due to vertical transmission through their mothers.

### Data analysis

The captured questionnaires were then translated back into English from Sesotho and IsiZulu by independent translators. The data collected from the interview questionnaires and checklist were saved using a Microsoft Excel spreadsheet from Google Forms. Google Forms was the data entry tool, and Excel was employed as a management strategy to ensure that the data entered were correct and error-free. Captured and cleaned data were imported into the STATA software version 18 (StataCorp LLC, College Station, Texas, United States). The researcher applied descriptive statistics. For univariate analysis, one of the independent variables was analysed, such as diagnosis period or duration on ART, birth polymerase chain reaction (PCR) done, or prophylactic regimen. Data on the demographic characteristics of respondents were grouped and tabulated to give proportions. Logistic regression assessed the factors associated with non-adherence to the PMTCT programme. The PMTCT programme is an intervention, or a guideline aimed at reducing the prevalence of HIV in pregnant women and eliminating MTCT. An infringement of the PMTCT programme refers, therefore, to a failure to comply with the guidelines stipulated in the programme.^10^ Bivariate logistic regression was performed, and variables with a significant association with the level of adherence at a 0.05 significance level were entered into multivariate logistic regression analyses to find predictors of adherence to the PMTCT programme. Cross-tabulation determined the factors associated with non-adherence and the prevalence of seroconversion. An odds ratio (OR) at 95% CI and a *P*-value < 0.05 were considered significant.

### Validity, reliability and bias

The questionnaire has been validated through content, construct, and face validity. To ensure the validity of the content, the questionnaire was of high quality and correctly measured the objectives of the research by using experts in the field. The construct validity assessed whether standard questionnaire items were sufficient to measure the proportion of non-compliance, and identified the factors that contributed to non-compliance with the programme. Face and content validity were verified by experts’ judgment in ensuring that the instrument measured what it intended to measure and that the target population of HIV-positive women in the PMTCT programme was clear.

Reliability refers to the consistency of measurements and the answer should be approximately the same at the end of the test. To determine the feasibility of the study, a pilot study was conducted to test the questionnaire with 20 respondents meeting the same inclusion criteria at the different health facilities. These respondents were not included in the main study and the results were captured and analysed to improve data collection research quality.

Biases can be introduced by phrasing the interview question in such a way that the interviewer intentionally suggests the responses of the respondents. A questionnaire administered by a researcher can potentially introduce socially desirable biases due to stigma; this effect was minimised by asking neutral questions and using a random and multiple selection response. Information bias was minimised by conducting a structured, standardised questionnaire during the interview and using an interview guide to assist in the selection process. Potential recall bias, such as recalling past events, was recorded under the limitations of the study.

### Ethical considerations

Ethical clearance (reference no.: SMUREC/H/341/2022:PG) was obtained from the Sefako Makgatho University Research Ethics Committee (SMUREC) for the research to be conducted. The researcher further requested permission from the Gauteng Department of Health (reference no.: GP_202302_069) to conduct the research at health facilities in Soweto, Johannesburg. An informed consent form was provided to each participant after the purpose of the study had been explained. Participation was voluntary, and respondents could withdraw at any time. If a participant became distressed during the interview, they would be referred to see any available counsellors, social workers, or psychologists within the facility. Confidentiality was ensured and maintained using an electronic link available to the researcher only. The information was only shared with healthcare providers for further care if needed, and no personal identifiers were used. The supervisors can access the information for the purpose of the study. The consent form was scanned and saved electronically. Codes instead of the participant’s name were used for the questionnaires to maintain anonymity. Interviewing of the respondents was conducted in a safe, and a private consultation room provided by the clinic manager of that facility to ensure the safety of the participants.

## Results

A total of 341 mother-baby pairs were recruited and took part in the study. The median age was 34. The majority (*n* = 149; 43.70%) were unmarried. Two hundred and twenty-two (65.10%) of the women reached high school, and 60 (17.60%) respondents reached tertiary level, and 221 (64.81%) were unemployed. The majority of the women (*n* = 141; 41.35%) had two children. The socio-demographic profiles of the respondents are shown in Table 1.

**TABLE 1:** Socio-demographic characteristics of respondents on the prevention of mother-to-child transmission programme (*N* = 341).

Variables	*n*	%	Adherence level				*P* *
			Non-adherent		Adherent		
			*n*	%	*n*	%	
**Age (years)**	-	-	-	-	-	-	0.092
17–24	57	16.72	38	66.67	19	33.33	-
25–34	189	55.43	91	48.15	98	51.85	-
35–44	92	26.98	46	50.00	46	50.00	-
> 45	3	0.88	2	66.67	1	33.33	-
**Marital status**	-	-	-	-	-	-	0.000
Unmarried	149	43.70	62	41.61	87	58.39	-
Married	48	14.08	26	54.17	22	45.83	-
Cohabiting	95	27.86	45	47.37	50	52.63	-
Divorced	14	4.11	12	85.71	2	14.29	-
Separated	25	7.33	24	96.00	1	4.00	-
Widowed	10	2.93	8	80.00	2	20.00	-
**Highest education achieved**	-	-	-	-	-	-	0.000
Not attended	27	7.92	24	88.89	3	11.11	-
Primary school	32	9.38	17	53.12	15	46.88	-
Secondary school	222	65.10	99	44.59	123	55.41	-
Tertiary school	60	17.60	37	61.67	23	38.33	-
**Employment status**	-	-	-	-	-	-	0.698
Employed	120	35.19	64	53.33	56	46.67	-
Unemployed	221	64.81	113	51.13	108	48.87	-
**Parity**	-	-	-	-	-	-	0.014
One	116	34.02	73	62.93	43	37.07	-
Two	141	41.35	72	51.06	69	48.94	-
Three	59	17.30	22	37.29	37	62.71	-
Four or more	25	7.33	10	39.13	15	60.87	-

* , Chi-square test.

### Socio-demographic characteristics of babies on the prevention of mother-to-child transmission programme (*N* = 341)

About 70% (*n* = 239) of the babies were aged 1 – 26 weeks, with an average age of 6 weeks, mean of 26.5, and s.d. = 22.5. One hundred and seventy-six (51.61%) were boys and 165 (48.39%) were girls. Two hundred and nine (61.29%) were exclusively breastfed, while 108 (31.67%) were formula-fed. Birth PCR was done for 336 (98.53%), and two of these were found to be HIV infected (Table 2).

**TABLE 2:** Socio-demographic characteristics of babies on the prevention of mother-to-child transmission programme (*N* = 341).

Variables	*n*	%
**Age (weeks)**		
1–26	239	70.09
27–52	59	17.30
53–104	43	12.60
**Gender**		
Female	165	48.39
Male	176	51.61
**Feeding practice from 0 to 6 weeks**		
Exclusively breastfed	209	61.29
Formula	108	31.67
Mixed	24	7.04
**Birth PCR**		
Yes	336	98.53
No	5	1.47
**HIV diagnosed**		
Negative	328	96.19
Diagnosed at birth	2	0.58
Seroconverted during breastfeeding	11	3.23

PCR, polymerase chain reaction.

### Factors related with prevention of mother-to-child transmission programme non-adherence

Several factors were identified to be significantly associated with non-adherence to the PMTCT programme. Experienced drug side effect, viral load suppression while on the PMTCT programme, missed appointments to PMTCT visits, and skipping of HIV medication were statistically significant, at a *P*-value of 0.000. Fear of stigma and discrimination was also found to be significant, at a *P*-value of 0.007, as was disclosure of maternal HIV status to a family member, at a *P*-value of 0.002 (Table 3).

**TABLE 3:** Clinical characteristics and adherence level on prevention of mother-to-child transmission programme (*N* = 341).

Variables	Level of adherence				*P* *
	Non-adherent		Adherent		
	*n*	%	*n*	%	
**Mother’s HIV diagnosed**	-	-	-	-	−0.152
Before pregnancy	118	50.86	114	49.14	-
During pregnancy	50	51.02	48	48.98	-
During delivery	4	66.67	2	33.33	-
Postpartum	5	100.00	0	0.00	-
**Maternal initiation of ART**	-	-	-	-	0.244
Before pregnancy	110	51.89	102	48.11	-
During pregnancy	58	49.57	59	50.43	-
After delivery	9	75.00	3	25.00	-
**Experienced side effects of drug**	-	-	-		0.000
Yes	133	65.84	69	34.16	-
No	44	31.65	95	68.35	-
**First antenatal booking**	-	-	-		0.103
Early	112	56.57	86	43.43	-
Late	62	44.93	76	55.07	-
Un-booked	3	60.00	2	40.00	-
**Viral load suppression while on PMTCT programme**	-	-	-		0.000
Always	7	63.64	4	36.36	-
Sometimes	134	58.65	68	41.35	-
Never suppressed	36	28.12	92	71.88	-
**Missed appointments to PMTCT visits**	-	-	-		0.000
Yes	94	85.02	19	15.08	
Never	83	36.40	145	63.60	
**Ever skipped taking HIV medication**	-	-	-		0.000
Yes	78	85.06	13	14.94	-
Never	99	39.60	151	60.40	-
**Fear of stigma and discrimination**	-	-	-		0.007
Yes	117	57.92	85	42.08	-
No	60	43.17	79	56.83	-
**Disclosure of maternal HIV status to family member**	-	-	-		0.002
Yes	100	45.66	119	54.34	-
No	77	63.11	45	36.89	-

ART, antiretroviral therapy; PMTCT, prevention of mother-to-child transmission.

* , Chi-square test.

### Perceptions of non-compliance with the prevention of mother-to-child transmission programme during pregnancy and breastfeeding

The results in Table 4 show that 213 (62.46%) of the women agreed that cultural practices affect long-term adherence to the PMTCT programme, while 56 (16.42%) disagreed. A significant portion of the women (*n* = 284; 83.28%) agreed that PMTCT drug intake benefits both mother and baby. Furthermore, 200 (58.65%) reported male involvement as being perceived to increase the effectiveness of PMTCT services and affect adherence positively. More than half (*n* = 183; 53.67%) of the women agreed that an individual lacking accurate health information can affect long-term adherence to the PMTCT programme.

**TABLE 4:** Perceptions questions and responses among HIV-positive pregnant and breastfeeding women towards the prevention of mother-to-child transmission programme.

Variables	Agree		Neutral		Disagree	
	*n*	%	*n*	%	*n*	%
Cultural practices affect long-term adherence to the PMTCT programme	213	62.46	72	21.11	56	16.42
It is tiresome to take PMTCT drugs every day	112	32.84	118	34.60	111	32.55
Taking PMTCT drugs benefits not only the mother but also the baby	284	83.28	44	12.90	13	3.81
Involving the male partner in care and support increases the effect on mothers adhering to the PMTCT programme, and effectiveness of PMTCT services	200	58.65	96	28.15	45	13.20
Lack of accurate health information	183	53.67	108	31.67	50	14.66

PMTCT, prevention of mother-to-child transmission.

### Knowledge influencing non-adherence to prevention of mother-to-child transmission programme during pregnancy and breastfeeding period

Table 5 presents the knowledge questions among HIV-positive women. Most women (*n* = 263; 77.13%) understood that an HIV-positive pregnant woman can transmit the virus to her unborn baby. Furthermore, 277 (81.23%) agreed with the statement that a woman may reduce the risk of transmitting the virus to their babies if the mother takes their antiretrovirals (ARVs). More than half of the women (*n* = 194; 56.89%) knew that missing a PMTCT drug dose would negatively affect long-term adherence to the programme. Some women (*n* = 268; 78.59%) believed adherence could reduce the risk of other opportunistic infections. Overall, 312 women (91.5%) were aware that the use of a condom can prevent the transmission of HIV during sex with an HIV-positive partner.

**TABLE 5:** Knowledge among HIV-positive pregnant and breastfeeding women towards the prevention of mother-to-child transmission programme.

The following characteristics affect long-term adherence to PMTCT programme	True		False		Unsure	
	*n*	%	*n*	%	*n*	%
Condom use can prevent HIV transmission during sex with an HIV-positive partner	312	91.50	13	3.81	16	4.69
Seropositive women can transmit HIV to their babies during pregnancy	263	77.13	32	9.38	46	13.49
HIV-positive women can reduce the risk of HIV transmission to their babies if they take PMTCT drugs	277	81.23	23	6.74	41	12.02
Skipping taking some of the PMTCT drugs does not affect the effectiveness of PMTCT care and support	70	20.53	194	56.89	77	22.58
Adhering to ARV drugs can reduce the risk of opportunistic infections	268	78.59	22	6.45	51	14.96

PMTCT, prevention of mother-to-child transmission; ARV, antiretroviral.

### Multivariate logistic regression for factors associated with non-adherence

The results in Table 6 show that being unmarried, the period of maternal HIV diagnosis and initiation on ART, unsuppressed viral load results, missed clinic appointments, side effects experienced, and getting tired of taking HIV medicines were all independently associated with non-adherence. Age, fears of stigma and discrimination, and the omission to take ARVs are not significantly associated with non-adherence.

**TABLE 6:** Multivariate logistic regression adjusted odds ratio of factors associated with non-adherence to prevention of mother-to-child transmission programme.

Independent variable	Adherence level				Univariate logistic regression			Multivariate logistic regression		
	Non-adherent		Adherent		OR	95% CI	*P*	aOR	95% CI	*P*
	*n*	%	*n*	%						
Age category	177	51.91	164	48.09	1.27	0.92–1.74	0.138	0.43	0.18–1.01	0.052
**Marital status**										
Cohabiting	45	47.37	50	52.63	Reference	-	-	-	-	-
Unmarried	62	41.61	87	58.39	5.31	3.40–8.46	< 0.001	5.73	1.74–18.90	0.004
Divorced	12	85.71	2	14.29	0.15	0.32–0.71	0.016	-	-	-
Separated	24	96.00	1	4.00	0.38	0.01–0.29	0.002	-	-	-
Widowed	8	80.00	2	20.00	0.23	0.05–1.12	0.068	-	-	-
Mother’s HIV diagnosed	177	51.91	164	48.09	0.76	0.53–1.10	0.143	0.05	0.01–0.38	0.004
**Unsuppressed viral load**										
Once	87	71.90	34	28.10	0.15	0.09–0.30	0.000	-	-	-
Seldom	41	61.19	26	38.81	0.25	0.13–0.50	0.000	0.18	0.05–0.70	0.012
Always	7	63.64	4	36.36	0.22	0.06–0.81	0.023	0.06	0.01–0.64	0.020
**Missed appointment**										
Once	54	78.26	15	21.74	0.16	0.08–0.30	0.000	0.23	0.70–0.80	0.017
Twice	36	92.31	3	7.69	0.05	0.14–0.16	0.000	0.07	0.08–0.62	0.016
More than three times	4	80.00	1	20.00	0.14	0.02–1.30	0.084	0.02	0.001–0.420	0.013
**Other factors**										
Maternal initiation on ART	177	51.91	164	48.09	1.30	0.82–2.15	0.024	13.51	1.80–101.83	0.012
Experienced side effects	177	51.91	164	48.09	4.16	2.63–6.60	0.000	4.24	1.42–12.61	0.009
Fear of stigma and discrimination	177	51.91	164	48.09	1.81	1.17–2.80	0.008	2.64	0.97–7.15	0.056
Getting tired of taking HIV medicines	177	51.91	164	48.09	0.78	0.60–1.02	0.067	0.33	0.20–0.63	0.001
Skipping taking ARVs	177	51.91	164	48.09	0.60	0.43–0.84	0.003	0.50	0.23–1.02	0.055

aOR, adjusted odds ratio; ART, antiretroviral therapy; ARV, antiretroviral; CI, confidence interval; OR, odds ratio; PMTCT, prevention of mother-to-child transmission.

Unmarried women had 5.7 times higher odds (aOR [adjusted odds ratio]: 5.73; 95% CI: 1.74 – 18.90; *P* = 0.004) of not adhering to the PMTCT programme than those cohabiting. In addition, respondents who reported having not experienced side effects were 4.24 times more likely to be adherent than those who reported them (aOR: 4.24; 95% CI: 1.42 – 12.61; *P* = 0.009). Women who feared stigmatisation and discrimination were 2.64 times more likely to be non-adherent to the PMTCT programme (aOR: 2.64; 95% CI: 0.97 – 7.15; *P* = 0.056) than those who were not fearful. Women starting ART during or after pregnancy are 13.5 times more likely to not adhere to ART as compared to women started before pregnancy (reference) (aOR: 13.5; 95% CI: 1.80 – 101.8; *P* = 0.012).

## Discussion

The study assessed the adherence, perceptions and knowledge of the PMTCT programme in mothers attending primary community healthcare centres with a high number of women living with HIV on the PMTCT programme. Despite the significant progress made globally in relation to PMTCT, the HIV/AIDS pandemic has continued to be a challenge to the maternal and paediatric services in SSA countries.^23^ Such challenges pose repercussions on other healthcare services that do not directly address the HIV- and AIDS-related conditions in SSA countries.^24^ Our study is similar to a study conducted by Nydal,^25^ which revealed that MTCT is still rated as a major public health challenge in many resource-poor countries.

Maternal socio-demographic factors were found to be related to the transmission of HIV from mother to child because of the poor uptake of HIV services in the PMTCT programme.^26^ Maternal socio-demographic factors that were found to be statistically significant with non-compliance with the PMTCT in our study include marital status, educational level, and parity. A high proportion of women who participated in this study had more than one child, and the majority were unmarried. After multivariate analysis using multiple logistic regression in this study, being unmarried (aOR: 5.73; 95% CI: 1.74 – 18.90; *P* = 0.004) was associated with a risk for non-adherence to the PMTCT programme. Some literature suggests that the utilisation of ANC services by unmarried women may be hampered by poor attitudes of health workers and poor access to antenatal clinics.^27^ In contrast, however, the study by Ogone^28^ found that married women (crude odds ratio [COR] = 1.95; 95% CI: 0.45 – 8.51) were more likely to achieve PMTCT compared with unmarried mothers.

In the study, the status of education was statistically significant. Literature indicates that there is a correlation between maternal education and HIV transmission. Four per cent of the mothers of children living with HIV were uneducated.^29^ This could be because women without education may not be aware of preventive measures to prevent HIV transmission from mother to child, resulting in inadequate treatment compliance. This is supported by an Ethiopian study which found that a maternal education level of Grade 1 – 6 was significantly and positively associated with HIV MTCT; moreover, mothers who reached secondary education were found to be more aware of maternal transmission to children and PMTCT.^26^ Other literature supports this by indicating that women with knowledge of PMTCT are 5.2 times more likely to be adherent to the PMTCT programme than women with poor knowledge.^30,31^ Though there is no universal way to assess knowledge of PMTCT, and the questionnaires used in various studies are different, the overall results of our study show that women respondents have good knowledge of different components of the PMTCT programme. Lack of knowledge is the strongest obstacle to compliance to ART.^32^ It is important that mothers who are enrolled in PMTCT have current knowledge so that they can promote their adherence in accordance with health beliefs.^33^ Consequently, insufficient knowledge about HIV/AIDS has had a negative impact on Ghanaian women who are HIV positive, as they were found to default on the treatment.^34^

Although the side effects of ARVs are reported to occur in the early stages of ART,^35^ most mothers in this study were enrolled in ART before becoming mothers. According to this current research, adverse drug side effects have been identified as a significant factor for poor participation in the PMTCT programme. This is consistent with a study conducted in the city of Hawasa, that revealed that lack of compliance with the drug results from a fear of the side effects, and it is associated with poor compliance with the B+ care option.^36^ Drug side effects are regarded as major challenges contributing to poor or non-adherence to treatment, reducing ARTs drug efficacy, and promoting virological failure, which increases the risk of vertical transmission among pregnant and breastfeeding women.^37,38^ Research conducted in northern Ethiopia has shown that proper advice on the side effects of ARV drugs is an important predictor of better compliance with PMTCT.^10^

Our study has found an association between non-adherence to the PMTCT programme and failure to achieve suppressed viral load. Mothers with high maternal viral load during pregnancy and breastfeeding run the risk of transmitting HIV infection to their babies. With the current high maternal HIV prevalence, to reduce MTCT rates to the level required to achieve the eradication of MTCT goal, South Africa must prioritise early initiation of ART and ensure that women maintain a viral load of 50 copies/mL or less throughout pregnancy and breastfeeding.^39^ In addition, monitoring performance against viral suppression targets and characteristics that influence viral suppression among pregnant women is essential for overcoming barriers to eradication of MTCT. Suboptimal viral suppression rates, despite a long duration on ART among these participants, indicates the need to strengthen the monitoring of viral load and treatment adherence throughout pregnancy.

The study found that there were still children diagnosed with HIV at birth and in the process of breastfeeding. This could be a result of the mother’s unabated viral load during pregnancy and breastfeeding. In addition, this can also be a sign of poor compliance with the PMTCT programme. Effective prevention of maternal infectious diseases requires mothers and their babies to receive PMTCT services that include ART and prophylaxis,^40^ to promote safe childbirth practices and appropriate infant feeding.^19^ Breastfeeding is encouraged because the literature shows that the combination of breastfeeding and ART significantly reduces the risk of transmission.^19^ Although women with HIV are encouraged to exclude breastfeeding, the study found that some children were fed with formula milk and others were mixed fed, which was not recommended by the PMTCT programme.

There are several factors that hinder HIV-positive women on eradication of MTCT service uptake, including clinical factors, perceptions, and knowledge. According to Manyi’s study,^41^ more than 95% of maternal-to-child HIV infections occur during pregnancy, labour, birth, or after breastfeeding. If ART is given to women during this time and to babies in the first weeks of life, the risk of MTCT can be reduced to less than 2%. Our study found that there were few infants who seroconverted at birth and breastfeeding; the purpose of the PMTCT programme was to eliminate the transmission of HIV to the infant.^42^

Since the implementation of the PMTCT programme in South Africa in 2006, the HIV MTCT rate has steadily declined, from 9.6% in 2008 to 0.9% in 2016. On the other hand, research in other African countries reported MTCT rates of up to 8.1%.

Despite the high level of HIV testing and ART adoption, it appears that MTCT has not yet been eliminated across southern African countries.^42^ Therefore, mothers should be encouraged to follow the PMTCT programme to reduce the risk of infection.

Although 56.57% of women complied with the early ANC booking, we still noted that some women booked late, and others did not attend antenatal services at all. Late ANC booking and no attendance at ANC delay the initiation of ART, and they are strong predicators of failure to achieve viral load suppression.^39^ Improvements in early booking at the ANC could improve the frequency of ANC visits and early ART initiation, which are good indicators of PMTCT programme compliance.

Appointments missed, skipping ARVs, and the ‘fatigue’ surrounding the taking of HIV drugs are considered behavioural limitations. This shows that the compliance with the PMTCT programme is complicated by many factors, affirming that non-compliance among HIV/AIDS mothers remains a crucial issue leading to only partial success of the programme. Consequently, poor compliance and treatment interruptions are labelled as issues limiting the effectiveness of PMTCT programmes in achieving the sustainable development goal (SDG) 3 to eradicate MTCT by 2030.^43^

Moreover, some of the difficulties relating to the non-adherence of respondents identified in the study to the PMTCT programme stem from factors including fear of stigmatisation and discrimination, and disclosure of the HIV status of the mother to family members. Fear of stigma and discrimination contributes to delays in access to medical facilities and delays in diagnosis, which could significantly contribute to the transmission of HIV from mothers to children. This is consistent with the findings of the survey, which revealed that 57.92% of respondents are afraid of stigmatisation and discrimination associated with the disclosure of their HIV status.^44^ Our survey also agrees with the research conducted in Vietnam, where it was reported that HIV-positive mothers are afraid of stigmatisation and discrimination, which led to their late diagnosis, poor quality of adherence, and poor access to antenatal and postnatal services.^45,46^

In contrast, 43.17% of respondents indicated that they were not afraid of stigmatisation or discrimination. Discrimination and stigmatisation are associated with poor drug use and lower willingness to seek medical treatment.^44^ Our study is conducted in a South African context, and shared the same sentiment that people living in low- and middle-income countries were at high risk of stigma associated with HIV/AIDS.^47^ The stigma associated with HIV for people living with HIV is mainly exacerbated by social support and deterioration of mental health functioning.^48^

On the other hand, stigma and discrimination related to HIV, as well as the disclosure of HIV status, are considered important elements in the continuous fight against HIV. People living with HIV are confronted with social stigma, making it difficult for them to disclose their HIV status.^34^ Poor or non-adherence, especially to ARTs, is associated with the lack of disclosure of an HIV-related condition by people living with HIV, whether to friends or family. In some cases, disclosure of HIV status is a stressful and complicated process that remains an important challenge and persistent barrier to effective HIV treatment.^49^ A small majority (51.32%) of respondents who revealed their HIV status said that disclosure was easy.

This result is consistent with those of the Cape Coast in Ghana study, which revealed that 76.6% of participants disclosed their status at least to one individual.^50^ Contrary to this study, a low disclosure rate of 33.3% of HIV status was reported in the eastern part of Ghana.^50^ Despite progress in HIV/AIDS management, fear of disclosure impedes access to health services and patient compliance.^49^ Furthermore, fear of disclosure affects PMTCT efforts and ART adherence among people living with HIV in many communities.^50^

Lastly, though 62.46% agreed that cultural practices affect long-term adherence to the PMTCT programme, 21.11% were neutral and 16.42% disagreed. Addressing cultural factors is the key to preventing HIV transmission from mother to child. Cultural characteristics influence the experience, decision and behaviour of women living with HIV, including the choice of infant feeding. Hence, it is not sufficient to provide antiretroviral medication and advice only to mothers living with HIV; the beliefs, attitudes, and practices of families and community members might have a negative impact on the adoption and adherence to the effectiveness of the PMTCT programme. To effectively eliminate MTCT of HIV in low-income countries, a series of steps must be taken, including the integration of socio-cultural factors into the PMTCT intervention programme.

On the other hand, 58.65% of respondents agreed that the inclusion of partners in the care and support of mothers living with HIV did not only influence the intention of the patient to remain on treatment, but also enhanced the effectiveness of the patient’s participation in the PMTCT programme. Men’s participation in ANC and PMTCT can increase adhesion to PMTCT strategies and have a positive impact on MTCT and infant survival rates.^51^ Furthermore, the involvement of men is related to increased uptake in the postnatal period, but it has minimal effect to influence neonatal and maternal health or increase PMTCT uptake.^52^ Although the involvement of men has increased social support and improved the health of babies and mothers, it is not sufficient to increase the adoption of PMTCT by women.^30^ Therefore, existing strategies to improve male participation should be studied and strengthened.

### Strengths and limitations

This complex study investigated the level of adherence, knowledge, and attitude to all components of the broader PMTCT programme in primary healthcare facilities in the context of the endemic maternal and child HIV transmission. Level of adherence was evaluated based on self-reports; thus, it may have contributed to a potential bias in recall.

During the interview, a questionnaire was administered by the researcher, and may have encouraged social desirability bias in responses because of a lack of anonymity. Socially desirable response bias may lead to inaccurate self-reporting, and respondents may be forced to answer questions in a way that is socially acceptable and not true, thereby affecting the validity of research results.

Although it is easier to apply, the use of a Likert visual analogy scale can lead to sensitivity that leads to over-exaggeration of compliance levels. This was observed during the interview, especially in an attempt to avoid the interviewer’s judgment, and the introduction of social desirability bias. Therefore, we believe that the study complements weaknesses with methodological strengths and that the conclusions are not very affected by potential limitations.

Thirdly, responses were self-reported, hence introducing a possibility of recall bias. However, this was minimised by verification of some responses from documented registers. Recall bias was also reduced by including only HIV-seropositive mothers who had children below the age of 2 years.

In addition, the research calculated the sample size using Raosoft software and used a convenience sample, but the proposed sample size was not achieved due to unforeseen reasons. Therefore, this study cannot be applied to other women living in HIV and in other settings.

## Conclusion

The study found that the level of HIV-positive women’s adherence to the PMTCT programme was suboptimal. As indicated in the findings of the study, poor or non-compliance with the PMTCT programme is a challenge, as it can be either intentional or unintended among mothers. It is important to understand the factors leading to the success of the PMTCT programme, because many factors affect compliance, including behavioural restrictions, counselling, stigmatisation and discrimination, knowledge, compromises, and cultural beliefs. Mothers lacking guidance on the PMTCT programme end up defaulting on compliance at any stage of the treatment. Therefore, measures targeting identified risk factors, such as stronger comprehensive advice and client education on the purposes of the PMTCT programme and possible side effects of drugs, disclosure of HIV status, and involvement of male partners, are mandatory for the elimination of MTCT and control of the epidemic. Success in preventing the spread of HIV from mother to child depends on the active role of health professionals in providing information on HIV to mothers, as well as the participation of various stakeholders.

In short, the risk of MTCT can be reduced through a series of evidence-based interventions in the PMTCT programme, which include a prevention strategy for MTCT with the aim of achieving a milestone of less than 2%.

The empowerment of pregnant and breastfeeding women to receive treatment will help to reduce elevated viral load and reduce the risk of vertical HIV transmission to babies. The continuous and successful implementation of the PMTCT programme and continuous support for adherence can reduce stigma and promote involuntary disclosure. In this respect, follow-up research is required to monitor the ongoing compliance with the PMTCT programme and its long-term impact on reducing the rate of transmission of HIV in mothers.
